# The mediating role of resilience and self-esteem between negative life events and positive social adjustment among left-behind adolescents in China: a cross-sectional study

**DOI:** 10.1186/s12888-019-2219-z

**Published:** 2019-08-01

**Authors:** Feifei Gao, Yuan Yao, Chengwen Yao, Yan Xiong, Honglin Ma, Hongbo Liu

**Affiliations:** 1 Department of Health Statistics, School of Public Health, Jinzhou Medical University, Jinzhou, 121000 Liaoning Province People’s Republic of China; 2 Department of Health Statistics, School of Public Health, China Medical University, Shenyang, 110122 Liaoning Province People’s Republic of China; 3 Medical College, Qingdao University, Qingdao, 266021 Shandong Province People’s Republic of China; 4 Middle School of Ying-Li Town, Heze, 274927 Shandong Province People’s Republic of China; 5 Hospital of Xi-He Town, Longquanyi district, Chengdu, 610107 Sichuan Province People’s Republic of China

**Keywords:** Left-behind adolescents, Social adjustment, Resilience, Self-esteem, Negative life events

## Abstract

**Background:**

In China, adolescents are frequently left behind by their parents. A great deal of scientific evidence demonstrates considerable psychological and social impacts that negative life events may have on adolescents who are left behind. While a direct relationship between negative life events and psychological and social effects has been observed, indirect effects have yet to be examined. Therefore, the objective of this study was to determine the association between negative life events and positive social adjustment and how resilience and self-esteem mediate this association.

**Methods:**

A cross-sectional study was carried out in the provinces of Shandong, Henan, and Sichuan in China. A questionnaire was distributed to 4716 left-behind adolescents in ten middle/high schools. We performed Bayesian estimations in structural equation modeling using the Markov Chain Monte Carlo algorithm to test our hypotheses.

**Results:**

Negative life events were significantly related to resilience (*r*_s_ = − 0.402), self-esteem (*r*_s_ = − 0.292), and positive social adjustment (*r*_s_ = − 0.239). Positive social adjustment was directly affected by resilience (*β* = 0.639) and self-esteem (*β* = 0.448). Negative life events were not only directly related to positive social adjustment (*β* = − 0.187, 95% credible interval: − 0.233 ~ − 0.139), but also showed an indirect effect on positive social adjustment (*β* = − 0.541, 95% credible interval: − 0.583 ~ − 0.501) through resilience (*β* = − 0.370) and self-esteem (*β* = − 0.171). The total effect of negative life events on positive social adjustment was − 0.728, where 74.31% was mediated by resilience and self-esteem. The indirect effect of negative life events on positive social adjustment through resilience and self-esteem was 2.893 times more than the direct effect.

**Conclusions:**

Resilience and self-esteem mediated most of the effect of negative life events on positive social adjustment. Interventions should be developed to improve the social adjustment of adolescents who are left behind, particularly the enhancement of resilience and self-esteem.

**Electronic supplementary material:**

The online version of this article (10.1186/s12888-019-2219-z) contains supplementary material, which is available to authorized users.

## Background

After several decades of fast economic growth resulting from the reform and launch of policies in China, the division between rural and urban Chinese with regard to living standards has grown substantially. Masses of rural people abandoned their hometown and poured into cities in search of better jobs and pay [1]. Most of the migrants were unable to take their families with them to cities due to their low incomes and poor living standards [2], so they had to leave their children back in their hometowns. These children are generally known as “left-behind children”. There are more than 61 million left-behind children living in China’s countryside [3]. The children get to spend very little time with their parents and some contact is usually by telephone. Adolescence is an essential period of socialization in a youth’s life [4, 5]. However, as much as 20% of adolescents around the world endure clear psychological complications or social disorders [6]. Left-behind adolescents (LBAs) are away from their parents for extended periods. Although they may benefit from their family having more money [7], the cost of social and psychological well-being must be heeded, which comes from the sacrifices these youths make with regard to physical and emotional intimacy. An earlier study showed that LBAs are susceptible to feelings of being deserted and unloved, with feelings of apprehension and confusion and worry, a situation that could have a negative impact on socialization [8].

Positive social adjustment (PSA) refers to being capable of seriously participating in social behavior and adjusting to the social situation at hand [9]. Previous investigations have demonstrated that separation from parents may have adverse effects on psychological and social development [10, 11]. In view of the parent-child separation that is unavoidable in China’s current social environment, the adverse effects of parent-child separation on PSA may be inevitable in a short time. Therefore, exploring influential factors of PSA is critical to psychological and social outcomes of LBAs. Negative life events (NLEs) are very important risk factors in the development of youths, and they have substantial effects on psychological and social end results [12, 13]. Although personality characteristics play a major role in the psychological and social outcomes of individuals, environmental experiences also contribute to these outcomes. LBAs experience increased levels of NLEs [14] and are often subjected to a more lax style of discipline, due to the absence of their parents. Of course, many LBAs are taken care of by family members and usually their grandparents, while some provide for themselves. The adverse effects of NLEs on PSA have been demonstrated in other populations [13, 15, 16]; however, up to now, this problem with NLEs in LBAs has been studied little. Accordingly, investigators are focusing on determining how to lessen the harmful effects of NLEs on PSA in these youths.

Resilience and self-esteem are important indicators of healthy psychological and social status. Resilience is defined as the ability of individuals to stay healthy and flexible in their lives, even when exposed to negative events [17]. Those who are highly resilient are more likely to have good social adaptability [18]. In addition, resilience is considered to be a mediator between social support and PSA [19], as well as a mediator between NLEs and subjective well-being among left-behind children [20]. Both the relationship between NLEs and resilience [13] and the relationship between resilience and PSA [19] have been researched in previous studies, but to our knowledge, the mediating effect of resilience between NLEs and PSA has not yet been confirmed. Basically, resilience is usually regarded as a comparatively stable and lasting feature, but under certain conditions, it may change. Accordingly, it is a dynamic phenomenon that can be altered at any moment [21]. It is possible that we may not be able to change the NLEs that the individual experiences. But if there is evidence demonstrating the mediating role of resilience between NLEs and PSA, improvement in PSA will likely be seen by intervening to enhance resilience in LBAs.

Meanwhile, self-esteem is also recognized as an important inner resource. It is the self-assessment that the individual makes and maintains [22]. Self-esteem is an inner mindset at the core of personality development and psychic equilibrium that adds throughout life to the development of adaptive processes [23]. People with low self-esteem might not have enough capacity to deal with day-to-day stress. Therefore, they are susceptible to emotional collapse and social maladjustment. Studies have showed that the level of self-esteem among LBAs is relatively lower than that of non-LABs [24] and that self-esteem is an important factor in reducing problems of social maladjustment [25, 26], which shows that individuals with high self-esteem will probably not experience social maladjustment. Moreover, self-esteem in adolescents is looked at as a mediator in the relationship between peer victimization and PSA [25] and between NLEs and delinquent behavior [27] as well. Although both the association between NLEs and self-esteem [28] and the relationship of self-esteem with PSA have been previously studied [25], self-esteem as a mediator in the relationship between NLEs and PSA among LBAs has not yet been confirmed to the best of our knowledge. Additionally, self-esteem may be improved through interventions [29, 30]. Therefore, if we can demonstrate the mediating effect of self-esteem between NLEs and PSA, PSA will also be improved through interventions in self-esteem among LBAs.

**Fig. 1:**
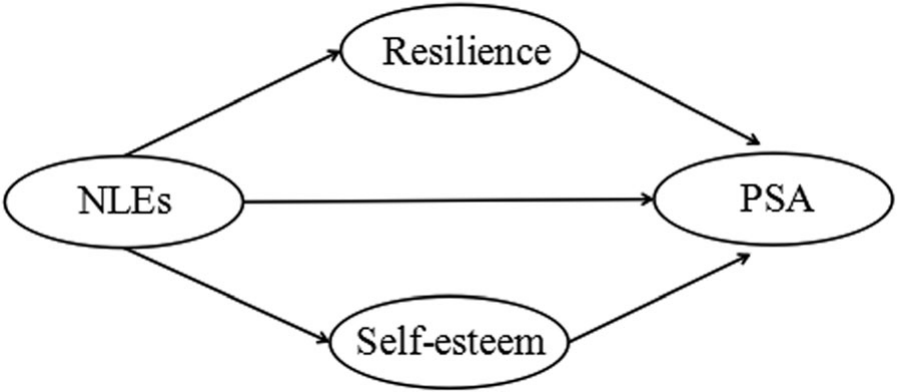
The theoretical hypothesis model on the relationship between NLEs, resilience, self-esteem and PSA. (PSA: Positive social adjustment; NLEs: Negative life events)

Taking into account the parent-child separation that is unavoidable in China’s current social environment, rapid enhancement of the NLE situation of LBAs may not be possible. Therefore, with demonstration of resilience and self-esteem playing mediating roles between NLEs and PSA, it is likely that PSA will be better with interventions to promote self-esteem and resilience among LBAs. Accordingly, this study aimed to analyze the influence of NLEs on resilience, self-esteem and PSA in LBAs. On the basis of our literature review, the theoretical hypothesis model was established as shown in Fig. 1. We proposed three hypotheses:

- Hypothesis 1: NLEs are negatively related to resilience, self-esteem and PSA.
- Hypothesis 2: Resilience and self-esteem are positively related to PSA.
- Hypothesis 3: Resilience and self-esteem mediate the relationship between NLEs and PSA.

## Methods

### Study design and sample

We carried out a cross-sectional study in April–May 2016, in a group of LBAs in the provinces of Shandong, Henan, and Sichuan in China. These three provinces are among those with the largest populations, and they are well known for sending workers to large cities in other parts of the country. There are many LBAs in these provinces, which can be characteristic of all of China.

Two-step random, stratified, cluster-based sampling was carried out. First, one city was randomly chosen in each of the provinces (Heze in Shandong, Zhoukou in Henan, Nanchong in Sichuan). We then randomly selected two middle schools and one high school in the countryside within each sampled city. If the selected school had < 200 students, one more school was selected. Seven middle schools and three high schools were included in our study. All individuals in the sampled schools were asked to participate in this study. We passed out all study-related materials to the students as a whole in the classroom with no teachers present, during a 30-min session.

Eventually, we tried to enroll 9675 students in this survey, where 355 individuals either refused to take part in the research or returned incomplete questionnaires, giving a total of 9320 (96.33%) in whom we sought to identify the LBAs. We classified the students as LBAs or non-LBAs by asking the question, “Did one or both of your parents migrate to another place because of work for at least 6 months?”. On the basis of the answer to this question, we categorized 4716 of the 9320 students (50.60%) as LBAs, all of whom were enrolled in this study (17.81% from Shandong, 44.15% from Henan, and 38.04% from Sichuan). The majority of the 4716 LBAs (65.65%) were in middle school, while 34.35% in high school. Their ages were 10–18 years (mean ± SD of 15.54 ± 2.24). Approximately, half were males (51.05%) and half were females (48.95%). A large majority of LBAs separated from both parents (70.06%), while 26.00% separated from the father and 3.94% separated from the mother. They were cared for by one parent (30.52%), grandparents (62.67%) or other relatives or even taking care of themselves (6.81%).

### Ethics statement

This study was conducted according to the Declaration of Helsinki. The study protocol was examined and approved by the ethics committee of Jinzhou Medical University [No: 2016–08], and it followed ethical standards. There was no sampling of biomarkers or tissue for analysis. Before administering any study-related questionnaires, all participants signed an informed consent form. Participation was completely anonymous, confidential, and voluntary. Participants could dropout from the study whenever they wanted.

### Measures

NLEs were measured by the Adolescent Self-Rating Life Events Check List (ASLEC), which is a 6-point Likert-type scale composed of 27 items in five areas: interpersonal relationship, study pressure, punishment, bereavement, and change for adaptation [31] (see Additional file 1: Table S1). The scale aims to assess the frequency and intensity of NLEs that participants experienced in the past half year. Responses are made based on a range, including 1 (did not occur), 2 (no effect), 3 (mild), 4 (moderate), 5 (severe) and 6 (extremely severe), which were scored as 0 (did not occur), 0 (no effect), 1 (mild), 2 (moderate), 3 (severe) to 4 (extremely severe). Higher scores represent greater stress related to NLEs. We performed a confirmatory factor analysis (CFA) to verify its factor structure using Bayesian estimation. The Markov Chain Monte Carlo (MCMC) algorithm was halted after generating 500 burn-in iterations and 44,500 analysis samples. The largest convergence statistics (*C.S.*) was 1.0008, which was below the 1.002 criterion that indicates acceptable convergence. In addition to the *C.S.* value, trace plots also suggested the convergence of the Bayesian MCMC method (see Additional file 2: Figure S1). The posterior predictive *p* (*ppp*) value was 0.43, indicating an acceptable fit of the model. The composite reliability of the scale was 0.8042 and Cronbach’s alpha was 0.813, with average variance extracted equal to 0.5142, suggesting a good internal quality of the model.

The Resilience Scale for Chinese Adolescent (RSCA) was used to measure resilience [32]. There are 23 Likert-type items with 4 dimensions (goal planning, emotional management, family support, and help seeking) (see Additional file 1: Table S2). Each of the items is scored on a 5-point Likert-type scale, with 1 indicating completely unmatched and 5 indicating exactly matched. High scores for the scale represent high levels of resilience. This scale has been evaluated with respect to reliability and validity [33]. The MCMC algorithm converges quite rapidly within 50,000 MCMC samples, while the highest *C.S.* was 1.0005 and the trace plots were ideal (see Additional file 2: Figure S2). The scale had an acceptable reliability (composite reliability was 0.7835 and Cronbach’s alpha was 0.868) and validity (average variance extracted was 0.4758), and there was an acceptable fit of the CFA model in our sample (*ppp* = 0.41).

Self-esteem was measured using the Self-esteem Scale (SES) [22] (see Additional file 1: Table S3). It comprised ten items, where each had 4 possible answers based on how much the respondent agrees (1 = strongly disagree, 4 = strongly agree), and the questionnaire has been shown to be valid in adolescents to determine their self-confidence and self-satisfaction [25, 34]. Total score for the 10 items indicates overall self-esteem, where higher scores mean greater self-esteem. The CFA model, in which the Bayesian MCMC method was used, reached an appropriate convergence criterion after 81,500 iterations. The highest *C.S.* was 1.0015, and visual inspection of the trace plot verified support for convergence (see Additional file 2: Figure S3). The *ppp* value was 0.38, which showed an acceptable model fit. The composite reliability (0.7976), Cronbach’s alpha (0.808) and the average variance extracted (0.4681) were also acceptable, indicating acceptable internal quality of the model.

The PSA scale was taken from the Social Adjustment Scale for Adolescents [35]. The scale is composed of 27 items that are determined on a 5-point Likert-type scale based on the extent of agreement with the participant’s experience (1 = strongly disagree, 5 = strongly agree) (see Additional file 1: Table S4). The scale assesses four factors of PSA, namely self-affirmation (8 items), pro-social tendency (7 items), efficiency of action (6 items), and positive coping (6 items). The CFA of the scale was performed using the MCMC algorithm before the analyses. The MCMC algorithm converged quite rapidly within 40,500 iterations, with the highest *C.S.* being 1.0006 and the trace plots ideal (see Additional file 2: Figure S4). The *ppp* value was 0.42, which suggested that the model fit was acceptable. The internal quality of the model was also acceptable, as the composite reliability (0.7610), Cronbach’s alpha (0.782) and the average variance extracted (0.4569) were ideal.

### Statistical analysis

Amos 20.0 software package (SPSS Inc., Chicago, IL, USA) and SPSS 16.0 (SPSS China Corp., Shanghai, China) were used for data analyses. The significance level was 0.05. Missing scale data were handled using the maximum likelihood method in AMOS 20.0. The categorical nature and the descriptive results of the survey items were considered, there was an evident lack of normal distribution for some variables [36], Spearman’s correlations were used to analyze the correlation between NLEs, resilience, self-esteem, and PSA. According to the theoretical hypothesis model, Bayesian structural equation modeling (SEM) was applied to explore the relationship between NLEs, resilience, self-esteem and PSA, which viewed any unknown quantity as a random variable and assigned it a probability distribution. MCMC methods were used to determine Bayesian estimates in SEM, in which the procedure was implemented in Amos 20.0. Bayesian analysis allows the estimation of variables on the basis of background knowledge to generate new information, and estimates can be obtained with MCMC sampling methods using the mean of the posterior distribution [37, 38]. In our study, the relationships between variables were not well known in LBAs, so objective Bayesian methods were used to establish the priors, that all priors could be considered poorly informative for the scale of the data [39]. Therefore, we used uninformative priors, which are believed to have little effect on model estimates [40], so that the data may be allowed to speak for themselves. By default, Amos applied a uniform distribution from −3.4 × 10^38^ to 3.4 × 10^38^ for each parameter [41]. Standardized estimates and 95% credible intervals (95% CI) for posterior estimates were determined. Stable parameter values were obtained when *C.S.* was under 1.002 and trace plots exhibited rapid up-and-down variation with no long-term trends or drifts [41]. Model fit was verified using Bayesian *ppp* values. The value of *ppp* of about 0.50 indicates a good-fitting model, while values of 0.3 to 0.7 may indicate an acceptable model [38]. In Bayesian inference, plausible reasoning was used to see whether a single hypothesis can be supported or to choose from several competing hypotheses [42]. Plausibility can be represented by likelihood or probability, that referred to the extent to which a hypothesis could be supported [42]. In this study, the probability that a regression coefficient is between the minimum and maximum bounds of the 95%CI was considered as a plausible association. The traditional effect size measures (*P*_*M*_ and *R*_*M*_) together with the standardized total effect, indirect effect and direct effect were used to measure the effect size of mediation model, where *P*_*M*_ refers to the ratio of the indirect effect to the total effect and *R*_*M*_ the ratio of the indirect effect to the direct effect. Before SEM, latent variables were established for variables with multiple items. Long ordinal scales or testlets are challenging for SEM [43]. The mean or sum of the scale were often used as an observational indicator of a latent variable when we incorporated a long scale into SEM [44]. This process was generally known as “parceling”. Preferable parceling conditions described so far include those with more than 12 items [45]. Parceling was most effective when original items had five response categories and there were two to six parcels were treated as observational indicators of latent variables [44]. In light of the above concerns, NLEs comprised five dimensions as their five indicators: interpersonal relationship, study pressure, punishment, bereavement, and change for adaptation. Resilience comprised four indicators, namely the four dimensions of the RSCA. PSA comprised four factors as its four indicators. Because the self-esteem scale has only 10 items, self-esteem used its 10 items as observed variables.

Before the theoretical hypothesis model was fitted, CFA was carried out to confirm the internal structural validity of the instruments using MCMC. Average variance extracted, composite reliability and Cronbach’s alpha were determined to assess the validity and reliability of the scales. Good validity refers to the average variance extracted value equal to or greater than 0.50, while values between 0.36 and 0.50 may be acceptable [46]. A composite reliability value equal to or greater than 0.60 indicates good reliability [47].

## Results

### Correlation between NLEs, resilience, self-esteem, and PSA

*Spearman’s* correlations were used to analyze the correlation between NLEs, resilience, self-esteem, and PSA. NLEs was significantly and inversely related to resilience (*r*_s_ = − 0.402, *p* < 0.01), self-esteem (*r*_s_ = − 0.292, *p* < 0.01), and PSA (*r*_s_ = − 0.239, *p* < 0.01). Resilience was significantly positively related to PSA (*r*_s_ = 0.520, *p* < 0.01). A positive relationship was also found between self-esteem and PSA (*r*_s_ = 0.521, *p* < 0.01).

### The relationship of NLEs, resilience, self-esteem and PSA

**Fig. 2:**
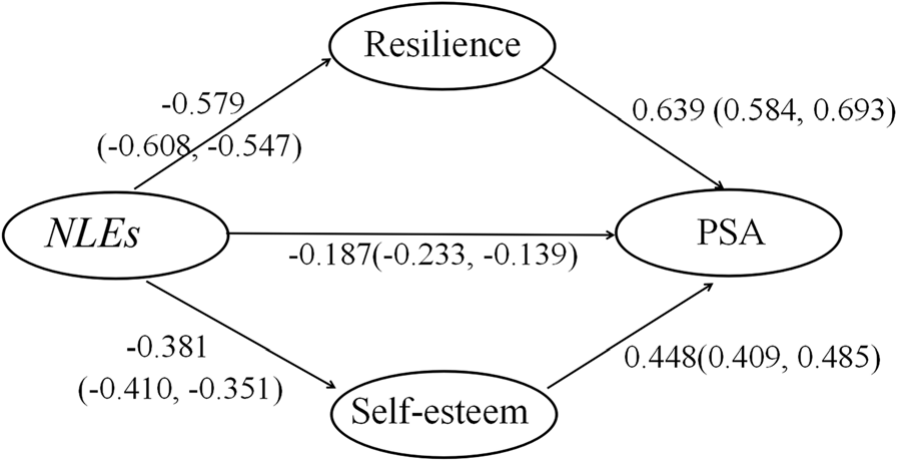
Bayesian estimation of the relationship between NLEs, resilience, self-esteem and PSA. (The standardized direct effects and 95% credible intervals between variables)

According to our theoretical hypotheses, the Bayesian approach was used for our SEM framework, where a normal distribution for a model’s parameters is not needed in the MCMC method. The hypothesized theoretical model met the conditions for stable parameter estimates after 92,090 iterations, while the convergence statistic was below 1.002 (*C.S.* = 1.0018). Furthermore, the Bayesian standard error obtained for each parameter was below 0.03. In addition, visual inspection of trace plots supported the convergence (see Additional file 2: Figures S5 and S6). The probability of the proposed theoretical model described in Fig. 1 was acceptable, in view of the data (*ppp* = 0.43). The main results of Bayesian statistics are shown in Fig. 2 and Table 1.

**Table 1:** The Bayesian analysis of standardized total effect, direct effect and indirect effect

Variables	Standardized mean	Standard deviation	Standard error	95% Credible interval	C.S.
Direct effect					
NLEs --- PSA	−0.187	0.0238	0.0014	(−0.233, − 0.139)	1.0018
NLEs --- Resilience	−0.579	0.0160	0.0009	(−0.608, − 0.547)	1.0015
NLEs --- Self-esteem	−0.381	0.0153	0.0005	(−0.410, − 0.351)	1.0005
Self-esteem --- PSA	0.448	0.0195	0.0008	(0.409, 0.485)	1.0009
Resilience --- PSA	0.639	0.0281	0.0015	(0.584, 0.693)	1.0015
Indirect effect					
NLEs --- PSA	−0.541	0.0212	0.0012	(−0.583, − 0.501)	1.0016
Total effect					
NLEs --- PSA	−0.728	0.0160	0.0009	(−0.816, − 0.640)	1.0016

**Fig. 3:**
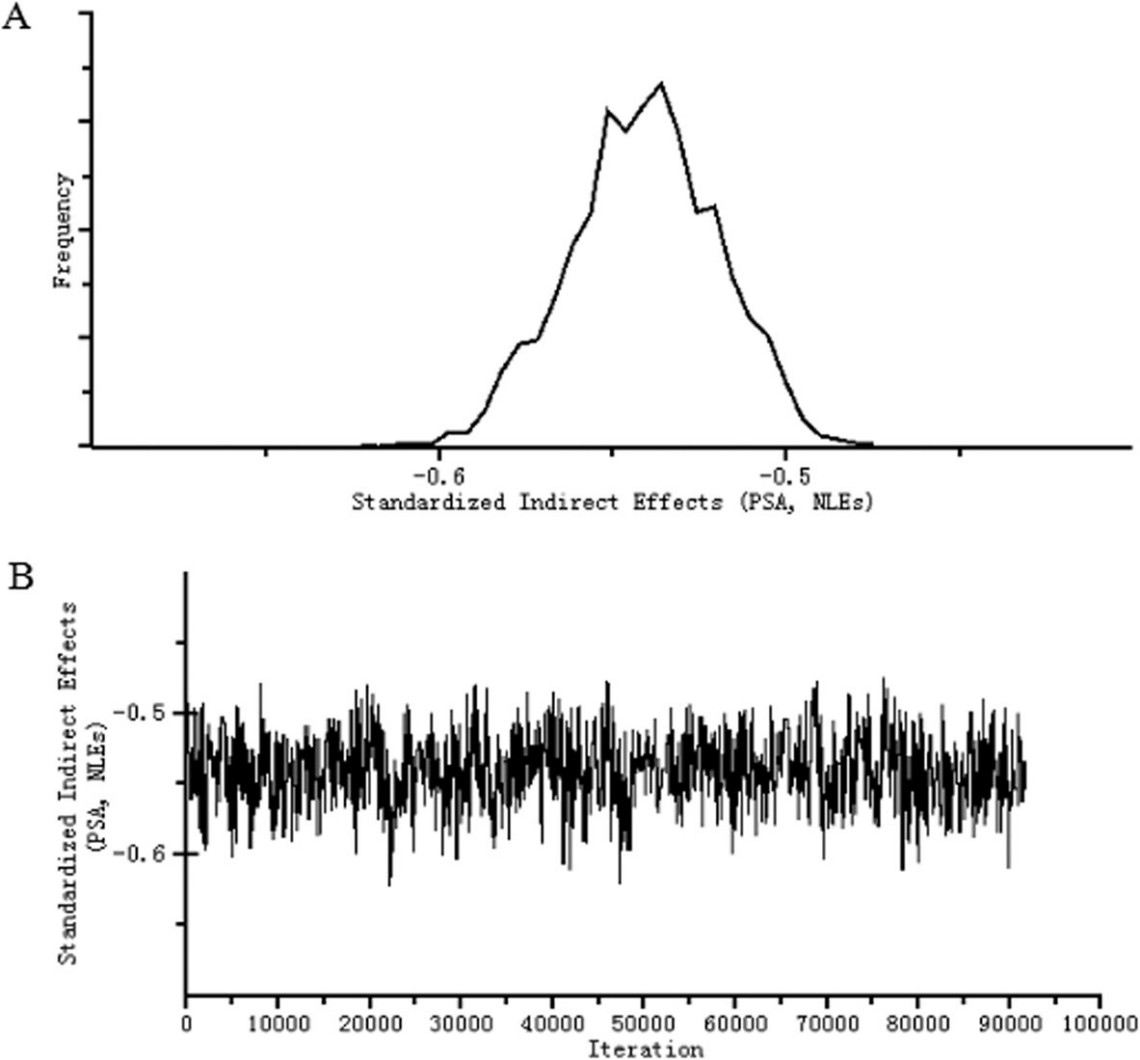
The posterior distribution of the standardized indirect effect of NLEs on PSA. (**a.** The frequency polygon of the distribution of the standardized indirect effect of NLEs on PSA; **b**.The trace plot of the standardized indirect effect of NLEs on PSA across the 92,090 iterations)

NLEs were negatively related to resilience (*C.S.* = 1.0015, *β* = − 0.579, 95% CI: − 0.608 ~ − 0.547) and self-esteem (*C.S.* = 1.0005, *β* = − 0.381, 95% CI: − 0.410 ~ − 0.351). Both self-esteem and resilience had a plausible positive direct effect on PSA (*C.S.* = 1.0009, *β* = 0.448, 95% CI: 0.409 ~ 0.485; *C.S*. = 1.0015, *β* = 0.639, 95% CI: 0.584 ~ 0.693, respectively). Additionally, NLEs were found to have a negative direct effect on PSA (*C.S.* = 1.0018, *β* = − 0.187, 95% CI: − 0.233 ~ − 0.139), and a plausible indirect effect was observed between NLEs and PSA (*C.S.* = 1.0016, *β* = − 0.541, 95% CI: − 0.583 ~ − 0.501), through resilience and self-esteem. Specifically, higher NLEs were associated with lower resilience (*β* = − 0.579, 95% CI: − 0.608 ~ − 0.547), and in turn, lower resilience of LBAs was related with lower PSA (*β* = 0.639, 95% CI: 0.584 ~ 0.693). The indirect effect of NLEs on PSA through resilience was − 0.370. Additionally, higher NLEs of LBAs were also associated with lower self-esteem (*β* = − 0.381, 95% CI: − 0.410 ~ − 0.351), and in turn, lower self-esteem was related with lower PSA among LBAs (*β* = 0.448, 95% CI: 0.409 ~ 0.485). The indirect effect of NLEs on PSA through self-esteem was − 0.171. Resilience and self-esteem significantly mediated the relationship between NLEs and PSA, yielding a plausible indirect relationship between NLEs and PSA (*β* = − 0.541, 95% CI: − 0.583 ~ − 0.501). The posterior distribution of the standardized indirect effect of NLEs on PSA is shown in Fig. 3.

According to the above findings, three paths were used to determine the effect of NLEs on PSA (See Table 2). First, the standardized direct effect of NLEs on PSA was − 0.187, accounting for just 25.69% of the total effect (− 0.728). Second, the standardized indirect effect of NLEs on PSA through resilience was − 0.370, which accounted for 68.39% of the indirect effect and 50.82% of the total effect (*P*_*M*_ =50.82%). In addition, the indirect effect of NLEs on PSA through resilience was 1.979 times more than the direct effect (*R*_*M*_ =1.979). Third, the standardized indirect effect of NLEs on PSA via self-esteem (− 0.171) was responsible for 31.61% of the indirect effect, and 23.49% of the total effect (*P*_*M*_ =23.49%). The indirect effect of NLEs on PSA through self-esteem was 0.914 times the direct effect (*R*_*M*_ =0.914). Therefore, 74.31% of the total effect of NLEs on PSA was mediated by resilience and self-esteem, and the indirect effect was 2.893 times more than the direct effect.

**Table 2:** The effect size of different paths in the relationship between NLEs and PSA

Path	Standardized effect	*P*_*M*_(%)	*R*_*M*_
NLEs --- PSA	−0.187	25.69	–
NLEs --- Resilience --- PSA	− 0.370	50.82	1.979
NLEs --- Self-esteem --- PSA	−0.171	23.49	0.914
Total	−0.728	100.00	2.893

## Discussion

Although the significance of parent-child separation to a youth’s physical and mental development has drawn increasing attention, issues about the underlying mediating mechanisms of resilience and self-esteem between NLEs and PSA in LBAs are still not well understood. This study examined the relationship of NLEs, resilience, self-esteem, and PSA in a large number of LBAs in rural China, and it explored the mediating role of resilience and self-esteem in the relationship between NLEs and PSA. Our main findings supported our hypotheses. NLEs were found to have a negative effect on PSA and indirect effects through resilience and self-esteem.

Consistent with previous results in other populations, NLEs were found to have a negative direct effect on PSA among LBAs, that is, those who experienced more NLEs had lower a level of PSA, confirming that NLEs are important influencing factors of PSA in LBAs [13, 48]. Additionally, those who were left behind by their parents at an early age experienced significantly more NLEs than others [14], suggesting potential psycho-social problems, where those who had more stressful encounters in life tended to be much more psychologically affected and were less able to adjust socially [48, 49]. Our study also showed that NLEs lowered LBAs’ resilience, which then was positively associated with LBAs’ PSA, meaning that resilience mediated the connection between NLEs and PSA. Therefore, LBAs with a higher level of resilience tended to have better social outcomes. These results are in line with previous research demonstrating that those with a higher level of resilience tend to cope more effectively with NLEs, resulting in fewer psychological and social problems in other populations [48, 50, 51]. This finding is in line with the notion that resilience is essential for achieving positive social outcomes [52], where it is negatively influenced by NLEs. Therefore, high resilience appears to be one of the mechanisms that explain why some LBAs can cope with many NLEs and are not troubled with social adjustment. As far as we know, this is the first report in the literature of such findings. Importantly, previous studies have shown that training not only significantly improves individuals’ resilience, but also effectively improves their social outcomes [53, 54]. Parent-child separation is unavoidable in China’s social system today, and its adverse effects on LBAs may be inevitable in a short time. We showed in the present study that NLEs were negatively connected with PSA, but this is not very practical in improving LBAs’ PSA by reducing NLEs, as we may not be able to change the NLEs that the individual experiences. Parent-child separation will eventually result in NLEs among LBAs. Current research suggests that targeted interventions to improve their resilience will contribute to enhancing PSA levels.

Meanwhile, consistent with previous studies [26–28], NLEs were found to have a negative influence on self-esteem, which has a clear influence on PSA. Self-esteem also acted as a mediator in the relationship between NLEs and PSA. This supports the notion that self-esteem is influential in achieving positive social outcomes, which are influenced by NLEs. The negative events that LBAs have experienced will decrease their positive psychological health, which will then lead to a higher risk of psychological and social problems. Having higher self-esteem may provide more resources to deal with daily stresses, making them stronger in averting social maladjustment, which supports the idea that self-esteem as an individual inner protective factor and important psychological resource that can be used to achieve positive social outcomes [55]. Teens often cannot evaluate themselves properly after experiencing too many NLEs, mainly in adolescence, an identity confusion phase, where low self-esteem makes them vulnerable to psycho-social disturbances [56–58], which explains why NLEs have an indirect effect on PSA through self-esteem. When self-esteem is raised, the negative effects of NLEs may be minimized to some degree, thereby enhancing PSA because of the positive effect of self-esteem on PSA. It is important to note that self-esteem is a dynamic rather static process [29, 59]. Interventions, which were developed to promote self-esteem, for example physical activity and programs based on mindfulness, have been beneficial in other groups, leading to positive changes in certain psychological and social outcomes [28, 59, 60]. It is probable that if these interventions are also effective among LBAs, they will be of great practical importance in enhancing PSA. Accordingly, we believe that there is an urgent need to develop and implement strategies for raising self-esteem in LBAs in China.

The results of our study enabled us to evaluate a set of complex relationships, rather than doing a direct analysis between variables. According to our results, resilience and self-esteem act as mediators in the relationship between NLEs and PSA. LBAs who experienced more NLEs tended to have lower levels of resilience and self-esteem, subsequently experiencing poor psychological and social outcomes. This approach provides insight into why LBAs who experience more NLEs exhibit problems with social adjustment. Consistent with previous research, higher levels of resilience and self-esteem may compensate for or offset the impacts of risk factors and reduce the negative consequences of adversity in children [56]. Together, these findings further suggest that resilience and self-esteem were positive tools to cope with daily stressful events and good support systems to improve the development of LBAs in our study. Therefore, a positive and feasible strategy to reduce the potential damaging effects of being left behind is to develop programs that enhance resilience and self-esteem in LBAs, resulting in an improvement not only in the lives of individuals but also in overall social functioning. Currently, much of the focus of the guardians of LBAs in China is on the teens’ academic progress and physical health, with many temporary guardians, parents, and even LBA managers (such as teachers, government agents, etc.) caring more about promoting their academics and physical health rather than their psycho-social function. Resilience and self-esteem are important psychological resources that can be developed [21, 29], and interventions, such as promoting physical activity, programs stressing mindfulness and training in mind-body skills, aimed at enhancing self-esteem and resilience have been beneficial in other groups [29, 30, 61, 62]. Thus, strategies for promoting resilience and self-esteem in LBAs in China must be developed and implemented as soon as possible. According to the findings of the present study, when resilience and self-esteem of LBAs are enhanced, the detrimental effects of NLEs may be offset somewhat and overall social functioning will be improved.

In view the above analysis, our findings are of great importance in managing the welfare of LBAs. First, we showed that resilience and self-esteem may help in making use of psychological resources that could deal with NLEs and enhance PSA. These results allowed us to realize the importance of self-esteem and resilience in coping with the adverse effect of NLEs, helping teens to maintain a healthy and positive state of mind. Second, our findings also afforded a new perspective for LBA managers to minimize the harmful effects of NLEs on PSA by providing LBAs with training programs for the development of resilience and self-esteem (for example, mindfulness-based programs). Furthermore, we believe that the development and application of systematic interventions aimed at enhancing resilience and self-esteem in the future will improve psychological and social outcomes in general.

While our findings can be of great importance for the management of LBAs, the limitations of this study should not be ignored. Our study had a cross-sectional design, where it was not possible to determine causality, and data were obtained over an established period. A longitudinal study can better explore the relationship of NLEs, self-esteem, resilience, and PSA, which tracks the PSA of LBAs during their growth development. Furthermore, our data depended on self-reporting, which may have led to information bias. Other facts obtained from the at-home parents, caregivers, teachers, and peers should also be included in further studies. Furthermore, only rural students were involved in our research. Students from other areas, not just the countryside, should also be studied in the future. Finally, positive psychological resources were measured by resilience and self-esteem in our study. For instance, positive psychological resources include more than resilience and self-esteem such as other aspects, for example well-being measures. To be able to enrich our results, more psychological measures should be included in future studies. Regardless of these limitations, our study gives us preliminary and novel insights into the mechanisms responsible for the associations between NLEs, resilience, self-esteem, and PSA in LBAs.

## Conclusions

In conclusion, our study found that NLEs had a negative effect on resilience, self-esteem and PSA, while resilience and self-esteem had a positive effect on PSA. Resilience and self-esteem acted as mediators between NLEs and PSA. Most of the effect of NLEs on PSA was mediated by resilience and self-esteem, where the indirect effect was 2.893 times more than the direct effect. Our study affords initial insight into the mechanisms that have a strong influence on the relationship between NLEs, resilience, self-esteem and PSA in LBAs, a topic that has not been examined in previous studies. These results allow us to realize how important resilience and self-esteem are to LBAs and to support the notion that LBA managers need to develop interventions aimed at enhancing resilience and self-esteem in LBAs, so that they can deal successfully with NLEs, thereby maintaining a healthy and positive social status. Due to the limitations of a cross-sectional design as in our study, future longitudinal studies should be conducted to better elucidate the dynamic connection between NLEs, resilience, self-esteem, and PSA.

## Additional files

Additional file 1: **Table S1.** Adolescent Self-Rating Life Events Check List (ASLEC). **Table S2.** The Resilience Scale for Chinese Adolescent. **Table S3.** Self-esteem Scale. **Table S4.** The Positive Social Adjustment Scale for Adolescents. (DOC 155 kb)

Additional file 2: **Figure S1.** The trace plot of the factor loading of an indicator variable (study pressure) to a latent variable (NLEs). **Figure S2.** The trace plot of the factor loading of an indicator variable (emotional management) to the latent variable (resilience). **Figure S3.** The trace plot of the factor loading of an indicator variable (the fourth item) to the latent variable (self-esteem). **Figure S4.** The trace plot of the factor loading of an indicator variable (pro-social tendency) to the latent variable (PSA). **Figure S5.** The trace plot of the standardized direct effect of resilience on PSA. **Figure S6.** The trace plot of the standardized direct effect of self-esteem on PSA. (DOC 1677 kb)

## Data Availability

The data sets used and /or analyzed during the current study are available from the corresponding author on reasonable request.

## Abbreviations

ASLEC: Adolescent Self-Rating Life Events Check List
*C.S.*: Convergence statistic
CI: Credible interval
LBAs: Left-behind adolescents
MCMC: The Markov Chain Monte Carlo algorithm
NLEs: Negative life events
*ppp*: Posterior predictive *p*
PSA: Positive social adjustment
RSCA: The Resilience Scale for Chinese Adolescent
SEM: Structural equation model
SES: Self-esteem Scale
