# Supplementary material for: The mediating role of resilience and self-esteem between negative life events and positive social adjustment among left-behind adolescents in China: a cross-sectional study
Source: BMC Psychiatry. 2019 Aug 1;19:239. doi: 10.1186/s12888-019-2219-z (PMC6676624; doi:10.1186/s12888-019-2219-z)
Supplement: Supplementary file 1 — Table S1. Adolescent Self-Rating Life Events Check List (ASLEC). Table S2. The Resilience Scale for Chinese Adolescent. Table S3. Self-esteem Scale. Table S4. The Positive Social Adjustment Scale for Adolescents. (DOC 155 kb) [file 12888_2019_2219_MOESM1_ESM.doc]

**No. Study site： No. school**：  **No. student：**

**Questionnaire survey on adolescent psychological behavior**

Dear students：

Thank you very much for your participation in our research. The following questionnaire contains the basic demographic characteristics and some psychological behaviors. We hope you can carefully read each question, and answer it according to your own actual situation and feelings. Please answer independently, do not refer to other people's opinions. You should answer according to your first impression. The results of the questionnaire are for research purposes only and will be kept strictly confidential. Additionally, you could dropout from the survey whenever you don't want to continue.

Thanks for your participation.

**Basic demographic characteristics:**

1. Your gender： ① male ② female
2. Your birthday：
3. Your grade:

① grade 1 of middle school ② grade 2 of middle school ③ grade 3 of middle school

④ grade 1 of high school ⑤ grade 2 of high school ⑥ grade 3 of high school

4. Did one or both of your parents migrate to another place because of work for at least 6 months?

① yes (if yes, please continue to answer question 5 and question 6) ② no (if no, please turn to table 1)

5. Who have migrated to another place because of work for at least 6 months?

①both of parents ②only father ③only mother

1. Who take care of you after one or both of your parents go out?

①mother ②father ③grandparents ④other relatives ⑤taking care of myself ⑥ others

**Table S1. Adolescent Self-Rating Life Events Check List (ASLEC)**

(Please read each item carefully and consider whether you or your family have experienced any of the following events in the past half year. If the event occurred, please mark "√" in the corresponding cell according to the degree to which the event affected you. If the event did not occur, simply mark √ in the event "did not occur" column.)

| Negative life events | 1=did not occur | If happened，the degree of influence to you | | | | |
| --- | --- | --- | --- | --- | --- | --- |
|  |  | 2=no effect | 3=mild | 4=moderate | 5=severe | 6=extremely severe |
| 1、Be misunderstood. | 1 | 2 | 3 | 4 | 5 | 6 |
| 2、Be discriminated against or ignored by others. | 1 | 2 | 3 | 4 | 5 | 6 |
| 3、Failed the exam or poor test scores. | 1 | 2 | 3 | 4 | 5 | 6 |
| 4、Clashed with the classmates or friends. | 1 | 2 | 3 | 4 | 5 | 6 |
| 5、The rules of life changed markedly. | 1 | 2 | 3 | 4 | 5 | 6 |
| 6、 Did not like school. | 1 | 2 | 3 | 4 | 5 | 6 |
| 7、 Love didn’t go well or lose love. | 1 | 2 | 3 | 4 | 5 | 6 |
| 8、Long time away from family members with no reunion. | 1 | 2 | 3 | 4 | 5 | 6 |
| 9、Great pressure on study. | 1 | 2 | 3 | 4 | 5 | 6 |
| 10、Had a bad relationship with teacher. | 1 | 2 | 3 | 4 | 5 | 6 |
| 11、Had an acute or serious illness. | 1 | 2 | 3 | 4 | 5 | 6 |
| 12、Relatives or friends had an acute or serious illness. | 1 | 2 | 3 | 4 | 5 | 6 |
| 13、Relatives or friends died. | 1 | 2 | 3 | 4 | 5 | 6 |
| 14、Be stolen or lost something. | 1 | 2 | 3 | 4 | 5 | 6 |
| 15、Be humiliated in public. | 1 | 2 | 3 | 4 | 5 | 6 |
| 16、The family was in bad financial condition. | 1 | 2 | 3 | 4 | 5 | 6 |
| 17、There are conflicts within the family. | 1 | 2 | 3 | 4 | 5 | 6 |
| 18、Failed in expected appraise (such as merit student). | 1 | 2 | 3 | 4 | 5 | 6 |
| 19、Be criticized or punished. | 1 | 2 | 3 | 4 | 5 | 6 |
| 20、Transfer or drop out of school. | 1 | 2 | 3 | 4 | 5 | 6 |
| 21、[B](http://dict.youdao.com/w/be fined/" \l "keyfrom=E2Ctranslation)e fined. | 1 | 2 | 3 | 4 | 5 | 6 |
| 22、[E](http://dict.youdao.com/w/enrollment pressure/" \l "keyfrom=E2Ctranslation)nrollment pressure. | 1 | 2 | 3 | 4 | 5 | 6 |
| 23、Fight with others. | 1 | 2 | 3 | 4 | 5 | 6 |
| 24、Be beat and scold by parents. | 1 | 2 | 3 | 4 | 5 | 6 |
| 25、Your family puts pressure on you to study. | 1 | 2 | 3 | 4 | 5 | 6 |
| 26、Accidental shock or accident. | 1 | 2 | 3 | 4 | 5 | 6 |
| 27、Other negative life events。 | 1 | 2 | 3 | 4 | 5 | 6 |

**Table S2. The Resilience Scale for Chinese Adolescent.**

(Please mark "√" in the corresponding number according to your actual situation.)

| Items | 1=completely unmatched 2=relatively unmatched  3=not clear 4=relatively matched 5=exactly matched | | | | |
| --- | --- | --- | --- | --- | --- |
| 1. Failure always discourages me. | 1 | 2 | 3 | 4 | 5 |
| 1. I have difficulties in controlling my mood, such as unhappiness. | 1 | 2 | 3 | 4 | 5 |
| 1. I have a clear life goal. | 1 | 2 | 3 | 4 | 5 |
| 1. I tend to be more mature and experienced after a setback. | 1 | 2 | 3 | 4 | 5 |
| 1. Failure and frustration make me doubt my ability. | 1 | 2 | 3 | 4 | 5 |
| 1. When I meet unpleasant things, I can't find a suitable person to talk to. | 1 | 2 | 3 | 4 | 5 |
| 1. I have one or more friends of my own age who can relate my difficulties to them. | 1 | 2 | 3 | 4 | 5 |
| 1. My parents respect my opinion very much. | 1 | 2 | 3 | 4 | 5 |
| 1. When I am in troubles and need help, no one I can turn to. | 1 | 2 | 3 | 4 | 5 |
| 1. I usually make a plan and a solution when I faced with difficulties. | 1 | 2 | 3 | 4 | 5 |
| 1. I'm used to keeping things to myself instead of talking to people. | 1 | 2 | 3 | 4 | 5 |
| 1. My parents are like to meddle in my ideas. | 1 | 2 | 3 | 4 | 5 |
| 1. No one listens to what I say at home. | 1 | 2 | 3 | 4 | 5 |
| 1. My parents lack of confidence in me and provide little spiritual support for me. | 1 | 2 | 3 | 4 | 5 |
| 1. I will talk to others when I have difficulties. | 1 | 2 | 3 | 4 | 5 |
| 1. My parents never criticize me. | 1 | 2 | 3 | 4 | 5 |
| 1. I will concentrate all my energy, when faced with difficulties,. | 1 | 2 | 3 | 4 | 5 |
| 1. It usually takes me a long time to forget the unpleasant things. | 1 | 2 | 3 | 4 | 5 |
| 1. My parents always encourage me to do my best. | 1 | 2 | 3 | 4 | 5 |
| 1. I can adjust my mood very well in a short time. | 1 | 2 | 3 | 4 | 5 |
| 1. I set goals for myself to push myself forward. | 1 | 2 | 3 | 4 | 5 |
| 1. I'm in a bad mood and I don't want to talk about it. | 1 | 2 | 3 | 4 | 5 |
| 1. I always mood swings that I'm prone to ups and downs. | 1 | 2 | 3 | 4 | 5 |

**Table S3. Self-esteem Scale.**

**(Answer the question according to your actual situation and mark √ in the corresponding position.)**

|  | Items | 1=strongly disagree | 2=disagree | 3=agree | 4=strongly agree |
| --- | --- | --- | --- | --- | --- |
| 1 | I feel that I am a person of worth. | 1 | 2 | 3 | 4 |
| 2 | I feel that I have a number of good qualities. | 1 | 2 | 3 | 4 |
| 3 | All in all, I am inclined to think that I am a failure. | 1 | 2 | 3 | 4 |
| 4 | I am able to do things as well as most other people. | 1 | 2 | 3 | 4 |
| 5 | I feel I do not have much to be proud of. | 1 | 2 | 3 | 4 |
| 6 | I take a positive attitude toward myself. | 1 | 2 | 3 | 4 |
| 7 | On the whole, I feel very satisfied with myself | 1 | 2 | 3 | 4 |
| 8 | I wish I could have more respect for myself. | 1 | 2 | 3 | 4 |
| 9 | I certainly feel useless at times. | 1 | 2 | 3 | 4 |
| 10 | At times, I think I am no good at all. | 1 | 2 | 3 | 4 |

**Table S4. The Positive Social Adjustment Scale for Adolescents.**

(Answer the question according to your actual situation and mark √ in the corresponding position.)

|  | Items | 1=strongly disagree | 2=relatively disagree | 3=not clear | 4=relatively agree | 5=strongly agree |
| --- | --- | --- | --- | --- | --- | --- |
| 1 | I'm proud of some of the things that I do. | 1 | 2 | 3 | 4 | 5 |
| 2 | I think I am no worse than others. | 1 | 2 | 3 | 4 | 5 |
| 3 | I think I have many good qualities. | 1 | 2 | 3 | 4 | 5 |
| 4 | I think I am useful and indispensable. | 1 | 2 | 3 | 4 | 5 |
| 5 | I believe I can do what others can do. | 1 | 2 | 3 | 4 | 5 |
| 6 | I am pleased with my appearance. | 1 | 2 | 3 | 4 | 5 |
| 7 | I am full of hope for my future. | 1 | 2 | 3 | 4 | 5 |
| 8 | I feel good about myself. | 1 | 2 | 3 | 4 | 5 |
| 9 | I am willing to help others sincerely. | 1 | 2 | 3 | 4 | 5 |
| 10 | I can put myself in other people's shoes. | 1 | 2 | 3 | 4 | 5 |
| 11 | I believe most people do things in well intentions. | 1 | 2 | 3 | 4 | 5 |
| 12 | I like to share things with other classmates or friends. | 1 | 2 | 3 | 4 | 5 |
| 13 | I really like most people around me. | 1 | 2 | 3 | 4 | 5 |
| 14 | I think most people I work with are honest and trustworthy. | 1 | 2 | 3 | 4 | 5 |
| 15 | I often comfort and encourage my friends when they are sad or unhappy. | 1 | 2 | 3 | 4 | 5 |
| 16 | Once I decide to do something, I will do it well. | 1 | 2 | 3 | 4 | 5 |
| 17 | I can arrange my time well. | 1 | 2 | 3 | 4 | 5 |
| 18 | I follow through. | 1 | 2 | 3 | 4 | 5 |
| 19 | I'm efficient and do things well. | 1 | 2 | 3 | 4 | 5 |
| 20 | When I encounter difficulties, I take it as a test of myself. | 1 | 2 | 3 | 4 | 5 |
| 21 | Even when things go wrong, I still can make the right decision. | 1 | 2 | 3 | 4 | 5 |
| 22 | I can face all kinds of difficulties in life and study. | 1 | 2 | 3 | 4 | 5 |
| 23 | I always look on the bright side of bad things. | 1 | 2 | 3 | 4 | 5 |
| 24 | I feel I can handle most of the problems I meet. | 1 | 2 | 3 | 4 | 5 |
| 25 | I don't think failure is a terrible thing. | 1 | 2 | 3 | 4 | 5 |
| 26 | I can get happiness in my study. | 1 | 2 | 3 | 4 | 5 |
| 27 | I study very hard. | 1 | 2 | 3 | 4 | 5 |

Thanks again for your participation!

Investigator:

date：
