# Supplementary figures and images for: The mediating role of resilience and self-esteem between negative life events and positive social adjustment among left-behind adolescents in China: a cross-sectional study
Source: BMC Psychiatry. 2019 Aug 1;19:239. doi: 10.1186/s12888-019-2219-z (PMC6676624; doi:10.1186/s12888-019-2219-z)

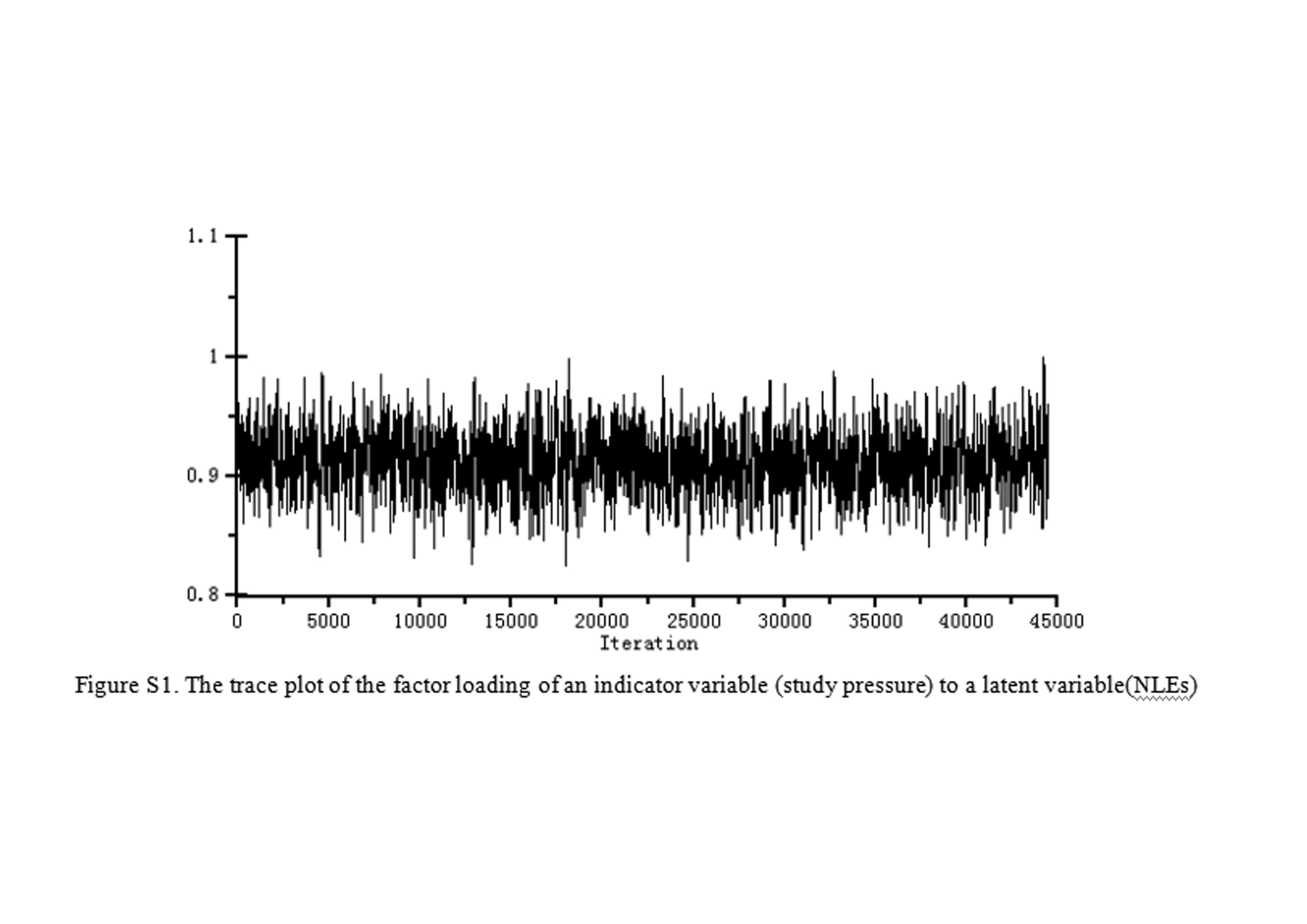


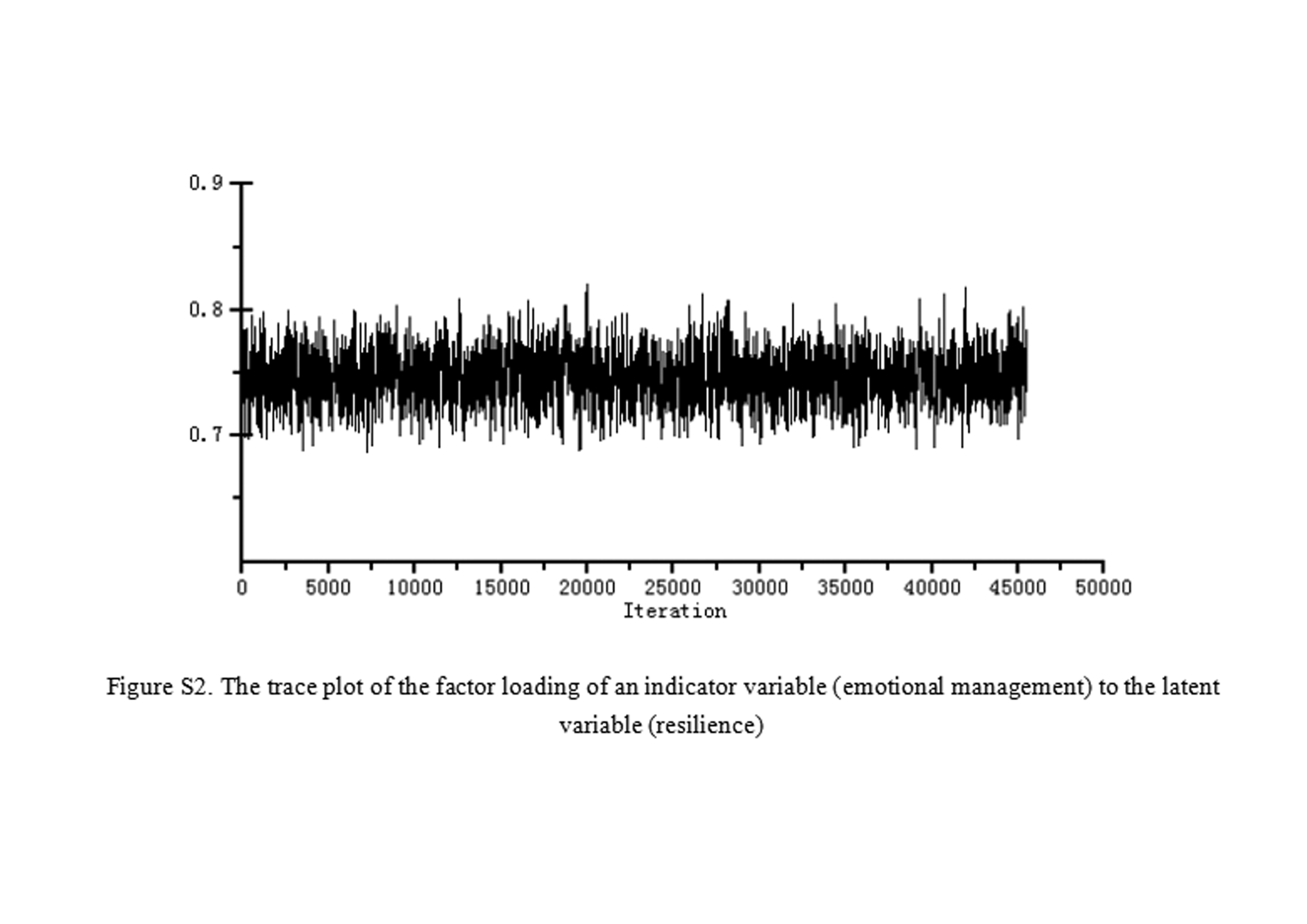


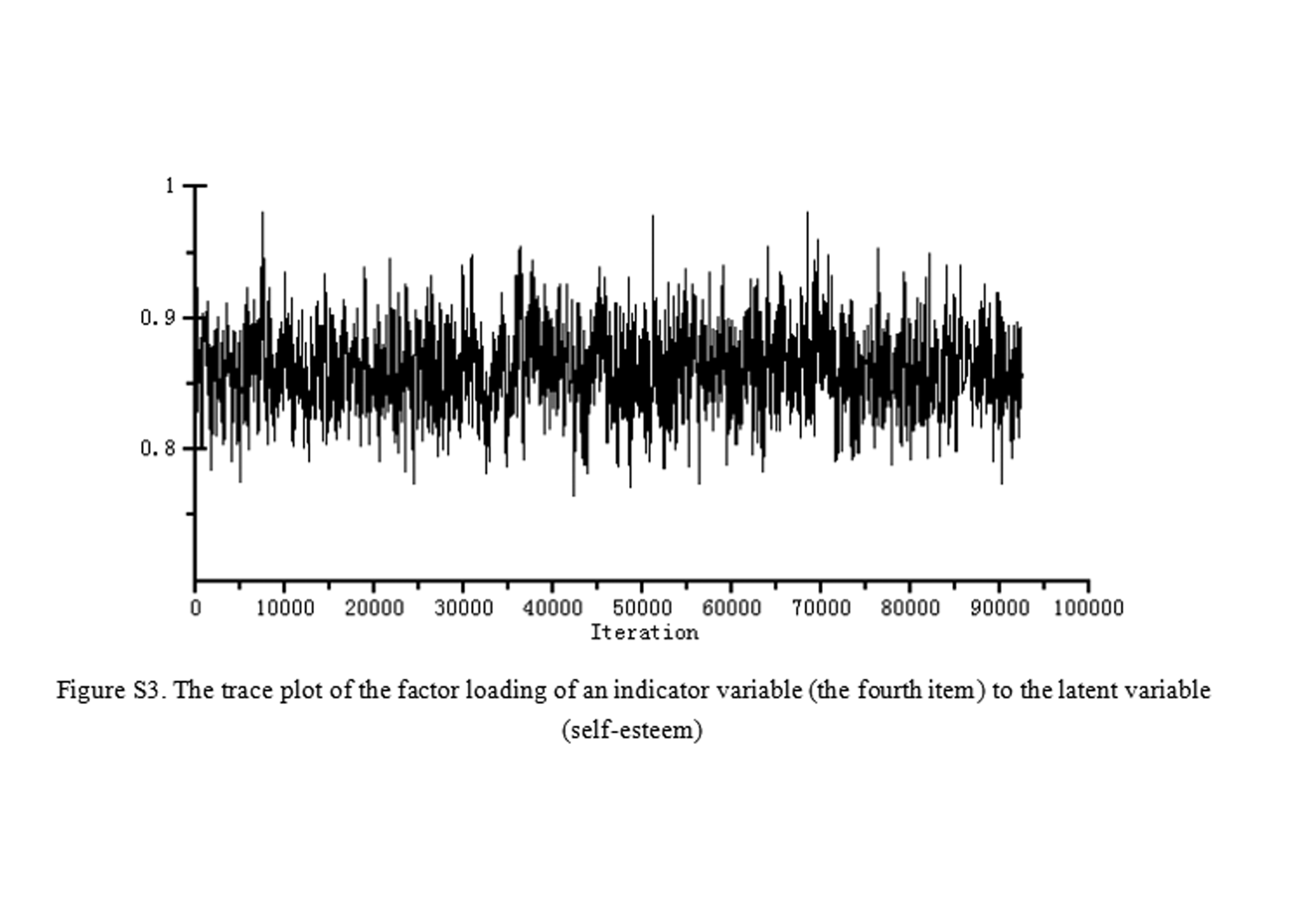


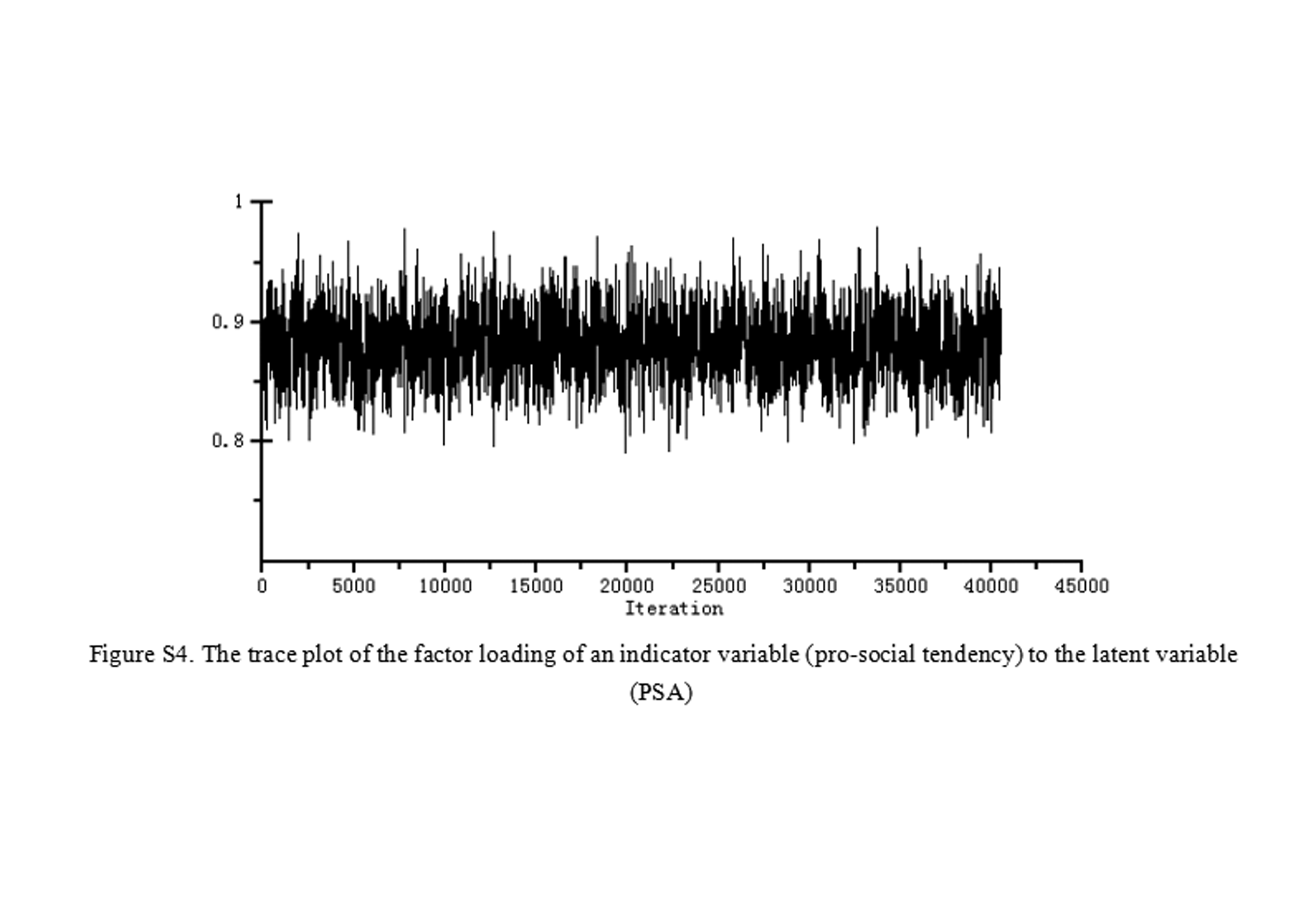


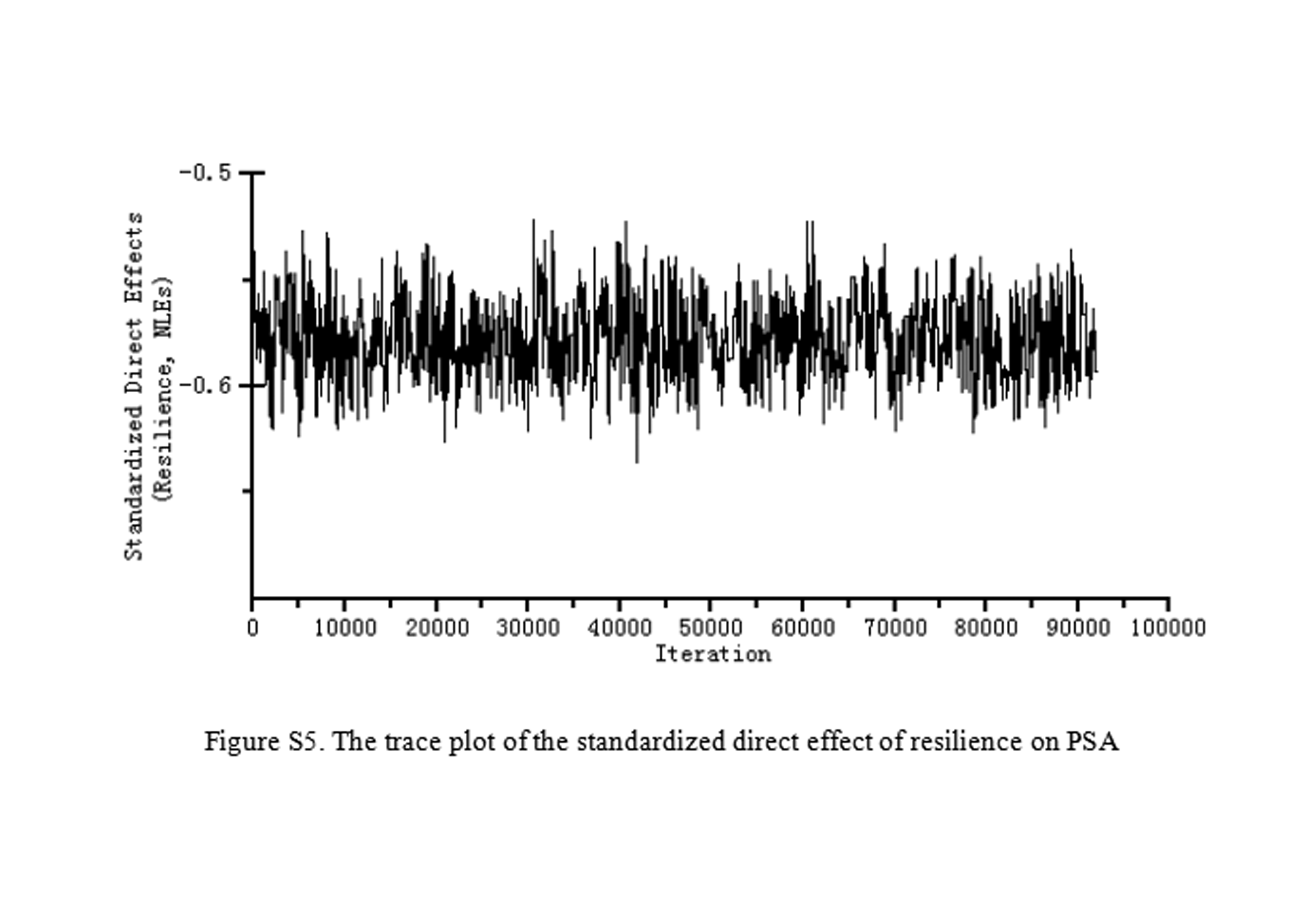


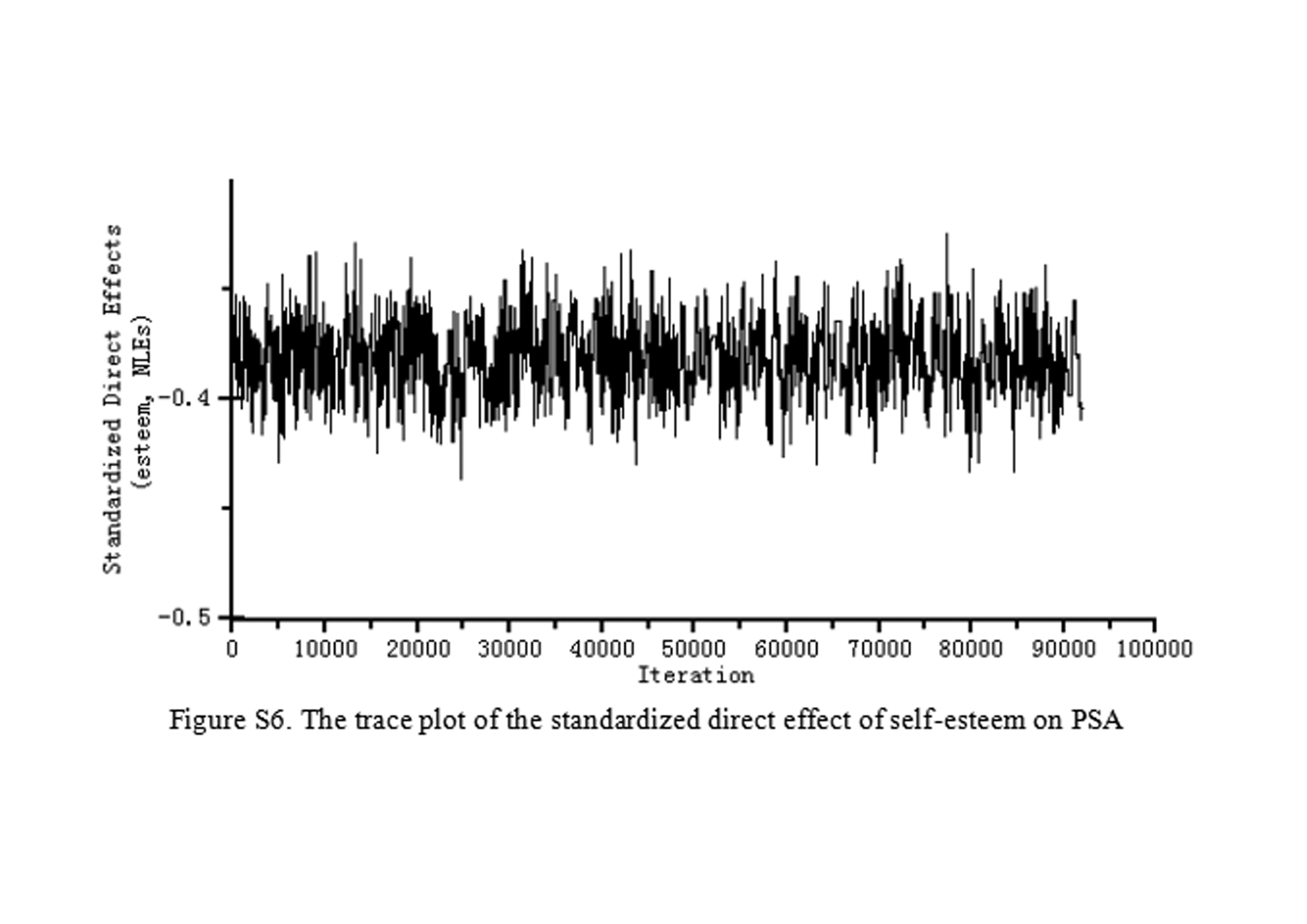

Supplement: Supplementary file 2 — Figure S1. The trace plot of the factor loading of an indicator variable (study pressure) to a latent variable (NLEs). Figure S2. The trace plot of the factor loading of an indicator variable (emotional management) to the latent variable (resilience). Figure S3. The trace plot of the factor loading of an indicator variable (the fourth item) to the latent variable (self-esteem). Figure S4. The trace plot of the factor loading of an indicator variable (pro-social tendency) to the latent variable (PSA). Figure S5. The trace plot of the standardized direct effect of resilience on PSA. Figure S6. The trace plot of the standardized direct effect of self-esteem on PSA. (DOC 1677 kb) [file 12888_2019_2219_MOESM2_ESM.doc]
